# Psychometric Properties of a Simplified Chinese Version of the Secondary Trauma Questionnaire in a Potentially Traumatized Study Sample

**DOI:** 10.3389/fpsyg.2020.00767

**Published:** 2020-05-15

**Authors:** Ya-jun Yan, Lichen Jiang, Mu-li Hu, Ling Wang, Xin Xu, Zhi-shuai Jin, Yu Song, Zhang-xiu Lu, You-qiao Chen, Na-ni Li, Jun Su, Da-xing Wu, Tao Xiao

**Affiliations:** ^1^Medical Psychological Center, The Second Xiangya Hospital, Central South University, Changsha, China; ^2^Research and Social Office, The Second Xiangya Hospital, Central South University, Changsha, China; ^3^Rehabilitation Department, Hunan Provincial People’s Hospital, The First Affiliated Hospital of Hunan Normal University, Changsha, China; ^4^Department of Science and Education, Zhuzhou 331 Hospital, Zhuzhou, China; ^5^National Clinical Research Center for Mental Disorders, Changsha, Hunan, China

**Keywords:** secondary trauma, secondary traumatic stress, psychometric properties, measurement, assessment

## Abstract

**Background:**

Screening for secondary traumatic stress (STS) is lacking in China. It is unclear whether Western models of STS can be adapted satisfactorily for use in non-Western regions. The 20-item Secondary Trauma Questionnaire (STQ) is a self-report measure of traumatic stress symptoms in individuals who have been influenced indirectly by suicide or violent injury of people important to the respondents.

**Methods:**

Here, we assessed the psychometric properties of a newly developed Chinese version of the STQ in a potentially traumatized sample (*N* = 875) composed of doctors, nurses, teachers, civic administration staff, and social workers in China. We performed reliability and validity analyses. Subsequently, we split the total sample into two subsamples for exploratory factor analysis (EFA) and confirmatory factor analysis (CFA) for measurement invariance analyses.

**Results:**

The full scale demonstrated good internal consistency (Cronbach’s α = 0.95–0.97), convergent validity, discriminant validity, and factorial validity. CFA affirmed a one-factor structure; the configural, metric, scalar, and strict invariances of the STQ were acceptable across genders.

**Conclusion:**

The present results indicate that the STQ is a reliable and valid self-report assessment for use with potentially traumatized people in China, and further supports the notion that the STQ is amenable to additional future cross-cultural adaptation.

## Introduction

The strong pathogenic impact of interpersonal trauma in posttraumatic stress disorder (PTSD) suggests that human interactions are particularly significant in triggering fear and other trauma-related responses (Charuvastra and Cloitre, 2008). A nascent and growing body of research suggests that caregiving professionals (Duffy et al., 2015; Denkinger et al., 2018; Greinacher et al., 2019) as well as witnesses and bystanders in the general public (Diehle et al., 2016; Anna, 2017) can develop secondary traumatic stress (STS) after experiencing close, continuous interactions with trauma victims that involve indirect exposure to graphic details of others’ traumatic experiences.

The most recently published diagnostic criteria for PTSD in the DSM-5 (Diagnostic and Statistical Manual of Mental Disorders, 5th edition) include the experience of repeated or extreme exposure to aversive details of a traumatic event while one is helping directly affected victims as a Criterion A stressor (American Psychiatric Association [APA], 2013). Thus, STS may represent a previously unrecognized clinically significant variant of PTSD. The symptoms of STS parallel the PTSD symptoms listed in the DSM-5, including intrusion, avoidance, altered cognition and mood, and functional impairment. Concerns have been raised that, in some cases of STS, responses to indirect trauma exposure may extend beyond the established cluster of PTSD symptoms to include moral distress, diminished professional self-efficacy, and feeling stigmatized (Sprang et al., 2019). Furthermore, given the high rate of comorbidity among traumatic stress, depression, and anxiety, persons experiencing STS may be at increased risk of experiencing depression and anxiety symptoms (Elwood et al., 2011).

The development of a thorough and clear understanding of STS has been hampered by conceptual inconsistencies. Notably, STS may have been identified under a variety of conceptual identifiers, including compassion fatigue, vicarious trauma, or burnout, depending on the context. Since Figley (1995) suggested that the term compassion fatigue may be a less stigmatizing term for STS phenomenon than other terms, the terms compassion fatigue and STS have been used largely interchangeably (Boscarino et al., 2010). However, the definition of STS is narrower and specific than that of compassion fatigue, which encompasses a strong empathic orientation and does not necessarily involve indirect exposure to a traumatic stressor (Bercier, 2013). Moreover, compassion fatigue caused by providing clinical services to traumatized populations may be an inherent feature of some mental health, medical care, and social work professions (Elwood et al., 2011). Beyond occupational exposure to STS, individuals may be at risk of STS when people they are close to in their personal lives suffer a traumatic event (Rodi, 2015; Anna, 2017). The concept of vicarious trauma involves a transformation of a care provider’s cognitive frame of reference, whereas the concept of STS encompasses social and emotional symptomology (Pearlman and Saakvitne, 1995; Jenkins and Baird, 2002). Furthermore, vicarious trauma is a cumulative process wherein empathic engagement with another’s traumatic experiences leads to the development of trauma symptoms in the empathizer, whereas STS encompasses a set of psychological symptoms acquired through indirect exposure to another person’s experiences (Baird and Kracen, 2006; Najjar et al., 2009). Finally, burnout exists in a wide variety of professions, whereas occupational STS is limited to contexts that involve a direct provision of care.

The psychometric properties and appropriate target populations for evaluating STS assessments have not been resolved. Eight STS-related self-report assessments were identified and divided into three types by research domain. The first type, including the Compassion Fatigue Self-Test for Psychotherapists (Figley and Stamm, 1996) and its derivative the Professional Quality of Life Scale (ProQOL; Stamm, 2005), are three-subscale questionnaires focused on compassion fatigue, with STS being recognized as a hyponym under the umbrella term compassion fatigue. In both of these instruments, only one subscale is used to measure STS, with concerns having been raised about the constructs of the other two subscales (e.g., Hemsworth et al., 2018; Heritage et al., 2018). A Chinese version of Compassion Fatigue Short Scale consisting of burnout and secondary trauma items was introduced and validated by Sun et al. (2011). The second type of assessments, including the World Assumptions Scale (Janoff-Bulman, 1989), Trauma Attachment and Belief Scale (Pearlman, 1996, 2003), and Secondary Trauma Self-Efficacy Scale (Cieslak et al., 2013), assesses changes in cognitive world-view schema, psychological need, and self-efficacy after indirect traumatic exposure. The third type, comprised of the Secondary Trauma Questionnaire (STQ; Motta et al., 1999), Secondary Traumatic Stress Scale (STSS; Bride et al., 2004), and Questionnaire for Secondary Traumatization (FST, originally *Fragebogenfür Sekundäre Traumatisierung*; Weitkamp et al., 2014), measures the frequency of PTSD-like symptoms in STS.

For the present study, we have adopted Figley’s original concept of STS, wherein STS is defined as “the natural and consequent behaviors and emotions resulting from knowing about a traumatizing event experienced by a significant other” (Figley, 1999, p. 10). This work is anchored to the third group of STS instruments above (STQ, STSS, and FST) to enable consideration of the results in the context of the latest research. All three of these instruments detect PTSD-like symptoms. The STSS is a 17-item scale that measures intrusion, arousal, and avoidance symptoms related to indirect occupational exposure to trauma. It has been shown to have good internal consistency (0.97; Bride et al., 2004) and has been validated across diverse populations (Makadia et al., 2017; Benuto et al., 2018). Currently, the newly proposed FST (Weitkamp et al., 2014) requires validity support and additional measurement invariance data. Particularly, both the STSS and the FST prioritize STS screening in people whose occupations involve working with traumatized people.

Given the original conceptualization of STS, the scope of professionals’ symptoms represented on the STSS or FST is quite narrow (Sprang et al., 2019). The STQ, which is the first proposed assessment to measure the frequency of STS symptoms, was derived from a delineation of the PTSD symptomology in the DSM-IV (American Psychiatric Association [APA], 1994). Continuing assessment and factor analysis of the STQ resulted in the modification of the original scale from 20 to 18 items by Motta et al. (2001), with two items being omitted (“I would have experienced horror or intense fear if I had their problems” and “I have disturbing recollections and intruding thoughts of their experiences”) to attain a more comprehensible factor structure. Following Motta et al. (2001) revision, a Farsi-language version of the STQ was demonstrated to have good internal consistency and discriminant validity in an Iranian study population (Ahmadi et al., 2016). Moreover, it has been reported to be suitable for broad application, including to mental health professionals, college students (Motta et al., 1999), Iranian child victims of warfare (Ahmadi et al., 2016), and relatives of adults living with cancer (Rodi, 2015). Motta et al. (2004) reported on the continued development of the STQ and the establishment of cutoff scores, such that scores in the range of 38–44 signify a moderate level of anxiety that may encompass problematic intrusive thoughts and avoidance symptoms and scores ≥45 signify a need for urgent intervention. Because the full 18-item version of the STQ could not be found (only the Abstract is available, Motta et al., 2001) and no response could be obtained following our attempts to contact the corresponding author, the 20-item STQ (Motta et al., 1999) was used in this study.

There is a lack of STS assessment instruments in China, where nearly 20% of the world’s population live. The aim of the current study was to evaluate the psychometric properties of a simplified Chinese version of the STQ in the potentially traumatized sample. The long-term goal of this work is to support efforts to make a simple, credible quantitative STS screening instrument available for application in the Chinese general public, including populations with an occupational risk of STS. To this end, we examined the factor structure of the Chinese STQ and its measurement equivalence across genders in a large, independent sample. Exploratory factor analysis (EFA) and confirmatory factor analysis (CFA) were employed to establish the factor structure of a Chinese version of the STQ.

## Materials and Methods

### Participants

The study was approved by the ethics committee at The Second Xiangya Hospital of Central South University. A cross-sectional study was used to investigate STS symptoms in a randomly sampled study cohort of 938 hospital, school, and Civil Affairs Bureau personnel from May 2016 to October 2018. Participants were recruited from three sources in Hunan province: (1) faculty at a secondary school in Changsha, where a student committed suicide, shocking the community (*N* = 105); (2) medical staff (doctors, nurses, and medical technicians) at a large polyclinic in Zhuzhou and Changsha, where a nurse had committed suicide recently and violent incidents affect medical staff regularly in outpatient services (*N* = 732); and (3) administrative staff and social workers (*N* = 101) at a grass-root level Civil Affairs Bureau, located in Changsha where a staff member committed suicide. At all three of these locations, authorities elevated the importance of employee mental health following a suicide or violent injury. Principals of the affected institution reported incidents of violence and suicide, and contacted our team for further mental crisis interventions. A precondition for participation was that the participants’ mental health was likely to have been affected indirectly by trauma, potentially by way of STS. The rationale for this sample selection was our interest in including a range of professions and types of exposure to graphic trauma details. We excluded 63 returned questionnaires due to missing demographic data or missing answers. Thus, a final sample of 875 participants were included.

### Measures

The questionnaire set included a brief survey to collect sociodemographic and work-related variable data as well as the following four self-report scales.

#### Secondary Trauma Questionnaire

Motta et al. (1999) developed the STQ to assess STS symptom levels in individuals who have been in extended close contact with a traumatized person. It has good reliability and validity across different populations (Motta et al., 2001). The scale consists of 20 items that are responded to on a 5-point Likert-like scale, ranging from 1 (rarely/never) to 5 (very often) with a higher score indicating a more severe STS symptom level.

In accordance with recommended procedures, including the back-translation method (Beaton et al., 2000; Chesterman, 2012), the original STQ was translated into simplified Chinese. First, an author of this study who is a clinical psychologist forward-translated the English STQ into Chinese. Second, the translation was reviewed by a committee comprised of six Chinese clinical psychologists and psychiatrists, each of whom evaluated the accuracy and applicability of the Chinese translation and expressions. The reviewer-modified Chinese translation was back-translated independently by a native Mandarin-speaking English major who was unaware of the purpose of the research. The back-translated English version was then inspected and amended as needed by the above review group. Finally, the original author examined the modified back-translated items and verified the content validity of the questionnaire.

#### Impact of Event Scale-Revised

Horowitz et al. (1979) developed the original Impact of Event Scale based on the DSM-III definition of PTSD to assess posttrauma symptoms. The revised version, the Impact of Event Scale-Revised (IES-R), produced by Weiss and Marmar (2007) has been used to probe the relationship between STS and PTSD. It has been shown to have good reliability and validity across versions as the screening tool of PTSD in various countries (e.g., Huang et al., 2006; Deng et al., 2016). Notably, the Chinese version used here has good reliability and validity (Huang et al., 2006). The IES-R consists of 22 items, distributed among three dimensions: intrusion, avoidance, and hyperarousal. Responses range from 0 (not at all) to 4 (extremely). The Cronbach’s α value of IES-R in this study was 0.97.

#### Depression Anxiety Stress Scale

The original Depression Anxiety Stress Scale (DASS) was compiled by Lovibond and Lovibond (1995). Wen et al. (2012) developed the Chinese version of the DASS employed in this study and confirmed that it has good reliability and validity for measuring the negative emotions of individuals in the past week. The scale consists of 21 items, distributed among three dimensions: depression, anxiety, and stress (7 questions in each dimension). The items are responded to via a four-level Likert-like scoring system: 0, does not apply to me at all; 1, applies to me to some degree; 2, applies to me to a considerable degree; and 3, applies to me very much. A higher DASS score indicates a higher negative emotional level. The Cronbach’s α for the DASS in this study was 0.96.

#### Posttraumatic Growth Inventory

Participants were administered with the Posttraumatic Growth Inventory (PTGI) (Tedeschi and Calhoun, 1996), a 21-item self-report instrument, to assess the perceived effects of traumatic events. The PTGI is comprised of five subscales (new possibilities, relating to others, personal strength, spiritual change, and appreciation of life). Respondents indicated how frequently they experienced feelings described in the items in the previous month using a six-choice, Likert-type response format ranging from 0 (did not undergo this change after trauma) to 5 (experienced this change after trauma). The Cronbach’s α value of the PTGI in this study was 0.96.

### Data Analysis

Statistical analysis was carried out in SPSS version 22.0 software. Multiple imputation was used to address item-level missingness as described previously (Beaton et al., 2000; Little and Rubin, 2002).

The data were split into two independent subsamples (1, *N* = 438; and 2, *N* = 437) using the random sample selection function in SPSS in preparation for EFA (performed in males and in females, respectively, with the varimax orthogonal axis method) and CFA to establish factor structure. Measurement invariance across genders was examined in Mplus (version 6.11). The factor analysis sample size was at least 10 times more than the number of variables (Maccallum et al., 2001). Non-normally distributed data estimation was conducted in Mplus software with the MLMV (maximum likelihood estimator with standard error and mean-variance correction chi-square test) method. In the CFA, the criteria for a satisfactory model fit were a root mean square error of approximation ≤0.08, a standardized root mean square residual ≤0.08, and comparative fit index (CFI)/Tucker–Lewis index values close to 0.90 (Browne and Cudeck, 1993; Hu and Bentler, 1999). Because chi-square testing is susceptible to sample size, we used CFI difference (ΔCFI < 0.01) to evaluate measurement invariance (Cheung and Rensvold, 2002).

Reliability was tested by calculating Cronbach’s α values and intraclass correlation coefficients. A measure that correlates moderately to strongly with related variables has convergent validity, whereas a measure that correlates poorly with variables unrelated to a construct has discriminant validity (Campbell and Fiske, 1959). Correlation analysis was conducted to evaluate convergent and discriminant validity. Specifically, convergent validity was assessed through correlational analysis of STQ scores with IES-R total scores and DASS total scores. Discriminate validity was assessed through the correlation analysis of STQ scores with the PTGI total scores.

One-sample *t*-tests were used to compare STQ scores across gender groups. A one-way analysis of variance (ANOVA) and least-significant difference (LSD) *post hoc* tests for multiple comparisons were performed to compare STQ scores across professional groups.

## Results

### Participant Characteristics

The demographic characteristics of the present study cohort, as a whole and by source, are summarized in Table 1. In total, 875 valid questionnaires were received. The cohort had a mean (*M*) age of 35.73 years with a standard deviation (SD) of 9.05 years (range, 19–63 years). Slightly more than two-thirds of the participants were women, and a majority had completed at least a bachelor’s degree; those that had not include elderly nurses and civil servants.

**TABLE 1:** Sample characteristics.

Characteristic		Source 1 *N* = 665	Source 2 *N* = 77	Source 3 *N* = 133	Total cohort *N* = 875	Total cohort (%)
Gender	Male	213	21	46	280	32
	Female	452	56	87	595	68
Age (years)	18–29	187	11	68	266	30.4
	30–39	237	28	37	302	34.5
	40–49	180	28	26	234	26.7
	50–63	61	10	2	73	8.3
Residence	Rural	361	43	69	473	54.1
	Township	64	8	16	88	10.1
	County seat	72	11	15	98	11.2
	Prefecture-level	140	11	29	180	20.6
	Provincial capital	28	4	4	36	4.1
Marital status	Single	141	19	28	188	21.5
	Married	502	58	97	657	75.1
	Widowed/divorced	22	0	8	30	3.4
Education	High/secondary	61	0	24	85	9.7
	College	205	37	27	269	30.7
	Bachelor	342	69	31	442	50.5
	Graduate degree	62	17	0	79	9.0
Profession	Doctor	244	0	0	244	27.9
	Nurse	347	0	0	347	39.7
	Medical technician	74	0	0	74	8.5
	Administrative	0	0	81	81	9.2
	Teacher	0	77	0	77	8.8
	Social worker	0	0	52	52	5.9
Professional title	Senior	18	4	4	26	3.0
	Sub-senior	94	14	18	126	14.4
	Middle	238	27	41	306	35.0
	Primary	213	22	49	284	32.5
	Other	102	10	21	133	15.2

### Comparison of Scores Based on Gender and Professional

Comparison across gender groups showed no significant differences in *M* total STQ scores (*t*_873_ = 0.96, *p* = 0.34, Hedges’ *g* = 0.07; male *M* ± SD = 41.19 ± 16.40, female *M* ± SD = 40.08 ± 15.47). A one-way ANOVA revealed a significant effect of occupational group on the STS level (*F*_6,__868_ = 2.78, *p* = 0.011, overall effect size *f* = 0.139), and LSD *post hoc* tests for multiple comparisons demonstrated that the *M* STS levels of doctors (38.54 ± 15.19, *p* = 0.004) and nurses (39.43 ± 15.48, *p* = 0.010) were significantly lower than that of teachers (44.51 ± 17.47).

### Factorial Validity

EFA (with varimax orthogonal axis rotation) was performed for the whole sample and separately for males and females in the subsample 1 (*N* = 438, 140 males and 298 females). For the whole sample, EFA results were suggestive of a one-factor structure with a Kaiser–Meyer–Olkin (KMO) value of 0.96 (exceeding the suggested value of 0.60). Bartlett’s test of sphericity yielded a significant result [χ^2^_190_ = 8467.31, *p* < 0.001], and the correlation matrix of the data was not zero, affirming that the data are suitable for factor analysis. EFA results of male and female samples also met these requirements (males KMO = 0.939, χ^2^_190_ = 2840.02, *p* < 0.001; females KMO = 0.961, χ^2^_190_ = 5856.65, *p* < 0.001). Matrix coefficients of factor loading are illustrated in Table 2; 64.4% of the variance (64% in males, 64.88% in females) could be explained by a single common factor in subsample 1.

**TABLE 2:** Matrix coefficients of factor loading and factor analysis for a one-factor structure solution.

Item	Varimax: one-factor			Commonalities		
	Total *N* = 438	Male *N* = 140	Female *N* = 298	Total *N* = 438	Male *N* = 140	Female *N* = 298
1	0.735	0.800	0.738	0.578	0.645	0.621
2	0.770	0.792	0.750	0.586	0.638	0.711
3	0.778	0.777	0.815	0.640	0.647	0.691
4	0.797	0.813	0.808	0.654	0.752	0.656
5	0.849	0.871	0.829	0.711	0.777	0.769
6	0.822	0.813	0.823	0.671	0.768	0.680
7	0.796	0.792	0.795	0.628	0.689	0.718
8	0.842	0.809	0.865	0.714	0.654	0.795
9	0.845	0.833	0.867	0.726	0.761	0.777
10	0.804	0.802	0.785	0.625	0.682	0.681
11	0.846	0.865	0.836	0.715	0.769	0.703
12	0.754	0.659	0.771	0.526	0.702	0.603
13	0.743	0.686	0.753	0.530	0.645	0.574
14	0.822	0.858	0.821	0.695	0.767	0.743
15	0.773	0.777	0.799	0.624	0.773	0.760
16	0.760	0.763	0.797	0.612	0.665	0.810
17	0.798	0.757	0.824	0.641	0.683	0.699
18	0.814	0.815	0.804	0.653	0.695	0.705
19	0.782	0.805	0.784	0.627	0.661	0.667
20	0.849	0.874	0.834	0.720	0.763	0.703

CFA was conducted in preparation for examining gender invariance in the single-factor model of STQ responses in the subsample 2 (*N* = 437); levels of configural invariance, metric invariance, scalar invariance, and strict invariance of the STQ were determined across gender groups. All factor loads, observed variable intercepts, and residuals were free estimates without any restrictions on configural invariance. The goodness-of-fit indices were within acceptable ranges. Therefore, the uncorrected configural invariance model was accepted as a baseline model for other measurement equivalence tests in accordance with the literature (Maccallum et al., 1992). When the factor load of the corresponding observed variable was assumed to be equal between genders in the metric invariance model, the parameters changed slightly compared with the baseline model. When an equal intercept of observed variables condition was added to test the scalar invariance model, we obtained a △CFI < 0.01. Assuming equal residuals of the corresponding variables equal in strict equivalence invariance model testing, a △CFI of 0.005 indicated strict invariance verification. For more methodological details, see Vandenberg and Lance (2000). The full configural, metric, scalar, and strict invariance results are reported in Table 3.

**TABLE 3:** Goodness of fit indices for equivalence restriction of five kinds on one-factor models of the STQ with error theory (*N* = 437).

Model	χ^2^	*df*	RMSEA (90% CI)	CFI	TLI	SRMR	AIC	△CFI
CFA	519.91	170	0.069 (0.060–0.074)	0.882	0.868	0.045	17,703.031	–
Configured invariance	665.47	340	0.066 (0.059–0.074)	0.874	0.859	0.050	17,748.891	–
Weak invariance	681.43	359	0.064 (0.057–0.071)	0.875	0.868	0.058	17,728.457	0.001
Strong invariance	700.86	378	0.063 (0.055–0.070)	0.875	0.874	0.059	17,708.774	0.000
Strict invariance	709.07	398	0.060 (0.053–0.067)	0.880	0.885	0.060	17,706.588	−0.005

**df*, degrees of freedom; RMSEA, root mean square error of approximation; CFI, comparative fit index; TLI, Tucker–Lewis Index; SRMR, standardized root mean square residual; AIC, Akaike information criterion.*

### Reliability

Total Cronbach’s α of the Chinese version of the STQ was 0.95 (0.97 for males, and 0.96 for females), with a reliability intraclass correlation coefficient of 0.76 (0.76 for males, and 0.77 for females). The mean STQ total score was 40.44 (SD = 15.76). The item result means (±*SDs)* and item-total score correlation coefficients (reported in Table 4) indicated a strong STS agreement and good internal consistency.

**TABLE 4:** Pearson correlational analysis of item scores and total scores in STQ (*N* = 875).

Item	M ± SD	Correlation coefficient
1	2.15 ± 1.01	0.737**
2	2.19 ± 1.04	0.773**
3	2.06 ± 1.00	0.755**
4	2.06 ± 1.02	0.791**
5	2.07 ± 0.96	0.833**
6	1.90 ± 1.00	0.789**
7	1.85 ± 0.93	0.749**
8	1.89 ± 0.94	0.800**
9	1.93 ± 0.94	0.815**
10	2.08 ± 1.04	0.782**
11	2.02 ± 0.98	0.830**
12	1.89 ± 1.00	0.732**
13	1.94 ± 0.94	0.754**
14	1.88 ± 0.93	0.803**
15	1.91 ± 0.93	0.763**
16	2.00 ± 0.96	0.766**
17	1.94 ± 0.89	0.780**
18	2.25 ± 1.09	0.819**
19	2.36 ± 1.12	0.795**
20	2.07 ± 1.02	0.833**

**p < 0.01.

### Convergent and Discriminant Validity

As shown in Table 5, STQ scores correlated positively and strongly with IES-R total scores, as well as with the intrusion, avoidance, and hyperarousal dimension subscores of the IES-R at similar magnitudes. Additionally, STQ scores correlated with DASS total scores and all DASS subscale scores. The results of these correlation analyses affirm convergent validity.

**TABLE 5:** Spearman intervariable correlation analysis results.

Variable	1	2	3	4	5	6	7	8	9
1. STQ	1.000	–	–	–	–	–	–	–	–
2. IES-R: Total	0.697**	1.000	–	–	–	–	–	–	–
3. IES-R: Intrusion	0.658**	0.967**	1.000	–	–	–	–	–	–
4. IES-R: Avoidance	0.693**	0.960**	0.899**	1.000	–	–	–	–	–
5. IES-R: Hyperarousal	0.666**	0.965**	0.915**	0.880**	1.000	–	–	–	–
6. DASS: Total	0.426**	0.366**	0.358**	0.396**	0.319**	1.000	–	–	–
7. DASS: Stress	0.426**	0.370**	0.362**	0.403**	0.320**	0.971**	1.000	–	–
8. DASS: Anxiety	0.409**	0.348**	0.339**	0.377**	0.307**	0.939**	0.888**	1.000	–
9. DASS: Depression	0.414**	0.362**	0.354**	0.388**	0.317**	0.941**	0.877**	0.840**	1.000

**p < 0.01.

Meanwhile, we found that STQ scores had a weak negative correlation with PTGI scores (*r* = -0.09, *p* < 0.01). The PTGI subscales of personal strength and appreciation of life were found to be similarly correlated with STQ scores (*r* = -0.13, *r* = -0.14, *p* < 0.01). STQ scores did not correlate significantly with the remaining three subscales (relating to others, *r* = -0.03, *p* = 0.31; new possibilities, *r* = -0.02, *p* = 0.66; spiritual change, *r* = -0.03, *p* = 0.37). These correlational analyses support the discriminant validity of STQ items.

## Discussion

The purpose of this study was to examine the psychometric properties of STQ in a large number of potentially traumatized professionals and further verify the factor structure and measurement invariance of the STQ across genders.

In our factor analysis, we found that a single-factor solution best accounted for the pattern of correlations among the variables. Motta et al. (1999) suggested that all items except items 18 and 19 loaded on the factors of Intrusion Cognition and Avoidance Behavior. Hereafter, Motta et al. (2001) deleted items 18 and 19 to make the factor structure of the STQ easier to understand and interpret. Ahmadi et al. (2016) developed and validated the modified 18-item STQ (items 18 and 19 omitted) for their Iranian warfare child victim study based on Motta et al. (2001), and they suggested a one-factor structure for the 18-item version. The present results also support a single-factor structure, but with the full STQ (no items omitted). The single-factor structure of the 20-item STQ in the potential traumatic population could be consequent to the cultural and professional heterogeneity of our sample. Notwithstanding, the present STQ findings are generally consistent with the findings of previous factor analytic studies of the STQ.

We evaluated STQ measurement invariance across genders to assess the potential widespread utility of the STQ in clinical practice, especially for mental health applications. The one-factor model showed a moderately good fit in the CFA results for subsample 2, though this outcome should be confirmed in a replication study given the chi-square index value. Tests of measurement invariance indicated that the factor structure, factor load patterns, variable intercepts, and residuals were similar in men and women. To the best of our knowledge, our study was the first to test STQ measurement invariance of a Chinese STQ across genders. These findings support introducing the STQ as an STS assessment tool in China.

In addition to factorial evidence of validity, internal consistency was acceptable for the total scale. This result is largely consistent with the results obtained by Ahmadi et al. (2016) with a cohort of children affected by war, albeit with a higher Cronbach’s α = 0.95. Intraclass correlation coefficients have not been reported for the STQ previously. Here, our data demonstrated convergent validity with the IES-R, which measures PTSD symptoms, as well as with the DASS, which is used to assess depression and anxiety levels. We further showed that the STQ has discriminant validity relative to the PTGI, which measures positive changes following trauma. Our correlational analyses showed that STQ scores correlated more strongly with the IES-R than with the DASS (total and subscales scores), which implies that the STQ is better suited for assessing STS symptoms than emotional problems. These findings further support the supposition that STS may be directly related to PTSD with a similar symptom presentation.

In terms of gender difference, although women are less often exposed to potentially traumatic events than men generally, they have been reported to exhibit more traumatic symptoms consistent with the diagnostic criteria for PTSD (Tolin and Foa, 2006; Baum, 2015). In this study, we did not observe a significant difference in STS levels between males and females. This statistical similarity of STQ total scores across genders can be attributed to inherent characteristics of the groups rather than the assessment. The female sample was composed of mostly nurses, who provide frontline services in a variety of emergency situations for patients who are experiencing trauma or are critically ill. It is possible that posttraumatic symptom growth among women arose as a result of stressful work environments. Although the data for women are encouraging, the STS levels of nurses cannot be ignored (Duffy et al., 2015).

Regarding the influence of profession group, we found that teachers reported higher levels of STS than doctors and nurses, although doctors and nurses experience more traumatic events (life-threatening illness, sudden violent death or sudden accidental death, etc.) throughout their work days than do study participants in the other included occupations. It could be that teachers applying extraordinary personnel connection and investment in their work as educators may make them more susceptible to STS after a student’s suicide. This unexpected finding merits immediate attention, particularly with respect to the availability of mental health support for teachers.

Taken together, the results of the current study have methodological and clinical implications for the study of STS. First, generally, the findings of this study support the cross-cultural validity of the STQ and confirm that the STQ has excellent psychometric properties as an STS assessment instrument. Second, our findings support the use of the STQ as a clinical STS screening tool in both the general population and for professionals in China. Third, the present results indicate that the STQ is both practical and suitable for use in China.

Although the present study supports the psychometric utility of the STQ, it had two noteworthy limitations. First, the varying gender ratios across the occupation groups could have introduced gender biases into the results. Second, this study was only a cross-sectional study without test–retest reliability or longitudinal equivalence analyses. Future work should take account of sample gender-balancing and collection of longitudinal empirical data.

## Conclusion

Our study demonstrated satisfactory psychometric properties of a Chinese version of STQ, a brief, reliable, and psychometrically valid screening test for potentially traumatized people, including professionals. The validity of this test should be evaluated further in a wider range of subjects, including, for example, a randomized sample of firefighters and police officers. Our findings further suggest that the STQ is amenable to adaptation to different cultures given that it is easy to conduct and does not seem to be hindered by strong language barriers. It is hoped that the current findings contribute to the development of appropriate STS assessment.

## Data Availability Statement

The datasets generated for this study are available on request to the corresponding author.

## Ethics Statement

All the data were collected with approval by an Ethics Committee of the Second Xiangya Hospital of Central South University.

## Author Contributions

DW and TX conceived and designed the study. YY and DW performed the analysis and prepared the manuscript. All authors were involved in the study conduction. All co-authors contributed substantially to its revision and approved the final manuscript.

## Conflict of Interest

The authors declare that the research was conducted in the absence of any commercial or financial relationships that could be construed as a potential conflict of interest.
